# 3-Methyl-1-benzofuran-2-carbohydrazide

**DOI:** 10.1107/S1600536812013190

**Published:** 2012-03-31

**Authors:** Hatem A. Abdel-Aziz, Hazem A. Ghabbour, Suchada Chantrapromma, Hoong-Kun Fun

**Affiliations:** a Department of Pharmaceutical Chemistry, College of Pharmacy, King Saud University, PO Box 2457, Riyadh 11451, Saudi Arabia; b Crystal Materials Research Unit, Department of Chemistry, Faculty of Science, Prince of Songkla University, Hat-Yai, Songkhla 90112, Thailand; c X-ray Crystallography Unit, School of Physics, Universiti Sains Malaysia, 11800 USM, Penang, Malaysia

## Abstract

In the asymmetric unit of the title benzofuran derivative, C_10_H_10_N_2_O_2_, there are three crystallograpically independent mol­ecules, which are slightly twisted; the dihedral angle between the benzofuran ring system and the plane of the carbohydrazide unit is 8.64 (11)° in one mol­ecule, whereas the dihedral angles are 9.58 (11) and 6.89 (10)° in the other two mol­ecules. In the crystal, the three independent mol­ecules are linked to each other through N—H⋯N hydrogen bonds, forming a trimer. The trimers are further linked by weak N—H⋯O and C—H⋯O hydrogen bonds into a three-dimensional network. π–π inter­actions with centroid–centroid distances in the range 3.4928 (11)–3.8561 (10) Å are also observed.

## Related literature

For bond-length data, see: Allen *et al.* (1987 ▶). For background to and the bioactivity of benzofuran derivatives, see: Abdel-Aziz & Mekawey (2009 ▶); Abdel-Aziz, Mekawey & Dawood (2009 ▶); Abdel-Wahab *et al.* (2009 ▶); Dawood *et al.* (2005 ▶); Hu *et al.* (2011 ▶); Ryu *et al.* (2010 ▶); Ungwitayatorn *et al.* (2001 ▶). For related structures, see: Ma *et al.* (2010 ▶); Wang *et al.* (2011 ▶).
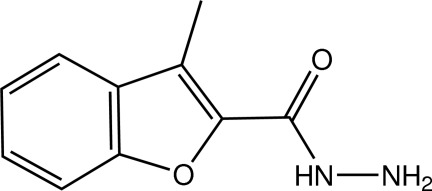


## Experimental

### 

#### Crystal data

C_10_H_10_N_2_O_2_
*M* *_r_* = 190.20
Monoclinic, 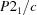

*a* = 10.9391 (4) Å
*b* = 18.1257 (6) Å
*c* = 14.1818 (5) Å
β = 94.157 (2)°
*V* = 2804.55 (17) Å^3^
*Z* = 12
Cu *K*α radiation
μ = 0.80 mm^−1^
*T* = 296 K
0.59 × 0.58 × 0.07 mm

#### Data collection

Bruker SMART APEXII CCD area-detector diffractometer
Absorption correction: multi-scan (*SADABS*; Bruker, 2009 ▶) *T*_min_ = 0.651, *T*_max_ = 0.946
19906 measured reflections
5271 independent reflections
3618 reflections with *I* > 2σ(*I*)
*R*_int_ = 0.048

#### Refinement

*R*[*F* ^2^ > 2σ(*F* ^2^)] = 0.045
*wR*(*F* ^2^) = 0.133
*S* = 1.00
5271 reflections
419 parameters
H atoms treated by a mixture of independent and constrained refinement
Δρ_max_ = 0.23 e Å^−3^
Δρ_min_ = −0.17 e Å^−3^

### 

Data collection: *APEX2* (Bruker, 2009 ▶); cell refinement: *SAINT* (Bruker, 2009 ▶); data reduction: *SAINT*; program(s) used to solve structure: *SHELXTL* (Sheldrick, 2008 ▶); program(s) used to refine structure: *SHELXTL*; molecular graphics: *SHELXTL*; software used to prepare material for publication: *SHELXTL* and *PLATON* (Spek, 2009 ▶).

## Supplementary Material

Crystal structure: contains datablock(s) global, I. DOI: 10.1107/S1600536812013190/is5101sup1.cif

Structure factors: contains datablock(s) I. DOI: 10.1107/S1600536812013190/is5101Isup2.hkl

Supplementary material file. DOI: 10.1107/S1600536812013190/is5101Isup3.cml

Additional supplementary materials:  crystallographic information; 3D view; checkCIF report

## Figures and Tables

**Table 1:** Hydrogen-bond geometry (Å, °)

*D*—H⋯*A*	*D*—H	H⋯*A*	*D*⋯*A*	*D*—H⋯*A*
N1*A*—H1*N*1⋯N2*C*	0.91 (2)	2.13 (2)	3.034 (2)	171.4 (17)
N1*B*—H2*N*1⋯N2*A*	0.93 (2)	2.15 (2)	3.081 (2)	173.1 (17)
N1*C*—H3*N*1⋯N2*B*	0.89 (2)	2.12 (2)	3.016 (2)	179 (3)
N2*A*—H2*N*2⋯O1*B*^i^	0.86 (2)	2.58 (2)	3.275 (2)	140 (2)
N2*B*—H4*N*2⋯O1*A*^ii^	0.93 (2)	2.33 (2)	3.176 (2)	150.4 (18)
N2*C*—H6*N*2⋯O1*C*^iii^	0.92 (2)	2.22 (2)	3.137 (2)	175.5 (17)
C6*A*—H6*AA*⋯O1*A*^iv^	0.93	2.55	3.444 (3)	162
C5*C*—H5*CA*⋯O1*B*^v^	0.93	2.59	3.333 (3)	137

Symmetry codes: (i) 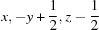; (ii) 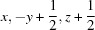; (iii) 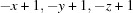; (iv) 
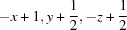
; (v) 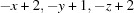.

**Fig. 1.**
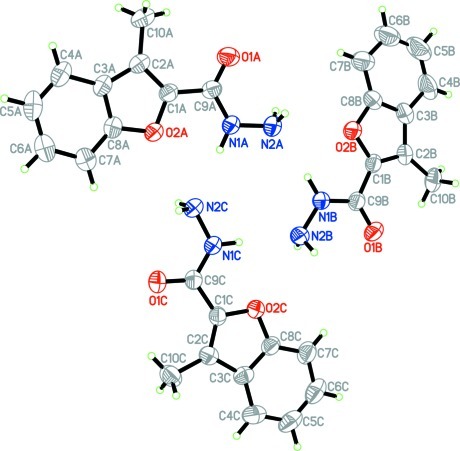
The asymmetric unit of the title compound, showing 40% probability displacement ellipsoids and the atom-numbering scheme.

**Fig. 2.**
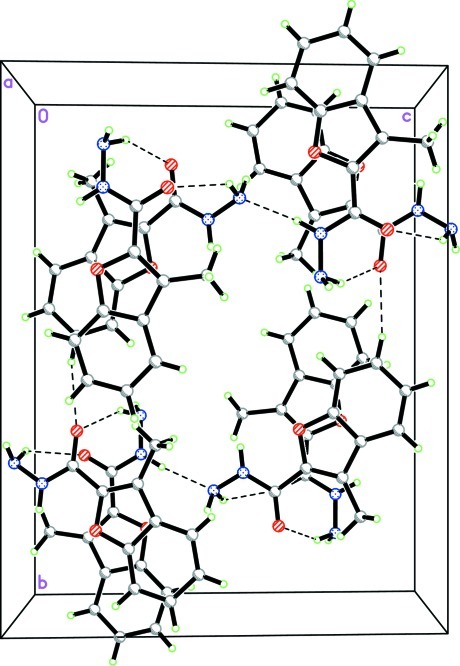
The crystal packing of the title compound viewed along the a axis. Hydrogen bonds are shown as dashed lines.

## supplementary crystallographic information

### Comment

Benzofuran derivatives have been reported to possess various biological activities such as antimicrobial (Abdel-Aziz & Mekawey, 2009; Abdel-Aziz, Mekawey & Dawood, 2009; Abdel-Wahab *et al.*, 2009), antifungal (Ryu *et al.*, 2010) and anti-inflammatory (Hu *et al.*, 2011) properties as well as being a non-nucleoside HIV-1 reverse transcriptase inhibitor (Ungwitayatorn *et al.*, 2001). We have during the course of our medicinal chemistry research reported the synthesis and bioactivity of benzofuran derivatives (Abdel-Aziz & Mekawey, 2009; Abdel-Aziz, Mekawey & Dawood, 2009; Abdel-Wahab *et al.*, 2009; ). The title compound (I) was synthesized and its crystal structure was reported.

There are three crystallographic independent molecules *A*, *B* and *C* in the asymmetric unit of (I) with differences in bond angles (Fig. 1). The molecule of the title benzofuran derivative, C_10_H_10_N_2_O_2_, is slightly twisted. The carbohydrazide fragment in molecules *A* and *B* are slightly twisted whereas it is planar in molecule *C* as indicated by the torsion angles of N2–N1–C9–O1 being -173.31 (17), -6.8 (3) and -179.64 (17)°, in molecules *A*, *B* and *C*, respectively. The dihedral angle between the mean plane through carbohydrazide fragment and the benzofuran ring is 8.64 (11)° in molecule *A* whereas they are 9.58 (11)) and 6.89 (10)° in molecules *B* and *C*, respectively. The bond distances agree with the literature values (Allen *et al.*, 1987) and are comparable with the related structures (Ma *et al.*, 2010; Wang *et al.*, 2011).

In the crystal packing (Fig. 2), the molecules are linked by N—H···N and N—H···O hydrogen bonds together with weak C—H···O interactions (Table 1). π–π interactions with the distances of *Cg*1···*Cg*4^i^ = 3.8561 (10) Å, *Cg*2···*Cg*7^iii^ = 3.4928 (11) Å, *Cg*_2_···*Cg*8^iii^ = 3.8179 (11) Å, *Cg*4···*Cg*7^vi^ = 3.7318 (10) Å and *Cg*4···*Cg*8^vi^ = 3.5891 (11) Å [symmetry code (vi) = 2-x, -1/2+y, 3/2-z] are also observed; *Cg*1, *Cg*2, *Cg*4, *Cg*7 and *Cg*8 are the centroids of C1A–C3A/C8A/O2A, C3A–C8A, C1B–C3B/C8B/O2B, C1C–C3C/C8C/O2C and C3C–C8C rings, respectively.

### Experimental

The title compound was prepared from the reaction of ethyl 3-methyl-2-benzofurancarboxylate with hydrazine according to the reported method (Dawood *et al.*, 2005). Single crystals of the title compound suitable for *X*-ray structure determination were recrystallized from ethanol by the slow evaporation of the solvent at room temperature after several days.

### Refinement

Hydrazide H atoms were located in a difference Fourier map and refined isotropically. The remaining H atoms were placed in calculated positions with C—H = 0.93 Å for aromatic and 0.96 Å for CH_3_ atoms. The *U*_iso_ values were constrained to be 1.5*U*_eq_ of the carrier atom for methyl H atoms and 1.2*U*_eq_ for the remaining H atoms. A rotating group model was used for the methyl groups.

### Crystal data

**Table:**

C_10_H_10_N_2_O_2_	*F*(000) = 1200
*M**_r_* = 190.20	*D*_x_ = 1.351 Mg m^−^^3^
Monoclinic, *P*2_1_/*c*	Cu *K*α radiation, λ = 1.54178 Å
Hall symbol: -P 2ybc	Cell parameters from 5217 reflections
*a* = 10.9391 (4) Å	θ = 4.0–69.9°
*b* = 18.1257 (6) Å	µ = 0.80 mm^−^^1^
*c* = 14.1818 (5) Å	*T* = 296 K
β = 94.157 (2)°	Plate, colorless
*V* = 2804.55 (17) Å^3^	0.59 × 0.58 × 0.07 mm
*Z* = 12	

### Data collection

**Table:**

Bruker SMART APEXII CCD area-detector diffractometer	5271 independent reflections
Radiation source: fine-focus sealed tube	3618 reflections with *I* > 2σ(*I*)
Graphite monochromator	*R*_int_ = 0.048
φ and ω scans	θ_max_ = 69.9°, θ_min_ = 4.0°
Absorption correction: multi-scan (*SADABS*; Bruker, 2009)	*h* = −13→12
*T*_min_ = 0.651, *T*_max_ = 0.946	*k* = −21→22
19906 measured reflections	*l* = −17→16

### Refinement

**Table:**

Refinement on *F*^2^	Secondary atom site location: difference Fourier map
Least-squares matrix: full	Hydrogen site location: inferred from neighbouring sites
*R*[*F*^2^ > 2σ(*F*^2^)] = 0.045	H atoms treated by a mixture of independent and constrained refinement
*wR*(*F*^2^) = 0.133	*w* = 1/[σ^2^(*F*_o_^2^) + (0.0825*P*)^2^] where *P* = (*F*_o_^2^ + 2*F*_c_^2^)/3
*S* = 1.00	(Δ/σ)_max_ = 0.003
5271 reflections	Δρ_max_ = 0.23 e Å^−^^3^
419 parameters	Δρ_min_ = −0.17 e Å^−^^3^
0 restraints	Extinction correction: *SHELXTL* (Sheldrick, 2008), Fc^*^=kFc[1+0.001xFc^2^λ^3^/sin(2θ)]^-1/4^
Primary atom site location: structure-invariant direct methods	Extinction coefficient: 0.00139 (18)

### Special details

**Table:**

Geometry. All esds (except the esd in the dihedral angle between two l.s. planes) are estimated using the full covariance matrix. The cell esds are taken into account individually in the estimation of esds in distances, angles and torsion angles; correlations between esds in cell parameters are only used when they are defined by crystal symmetry. An approximate (isotropic) treatment of cell esds is used for estimating esds involving l.s. planes.
Refinement. Refinement of F^2^ against ALL reflections. The weighted R-factor wR and goodness of fit S are based on F^2^, conventional R-factors R are based on F, with F set to zero for negative F^2^. The threshold expression of F^2^ > 2sigma(F^2^) is used only for calculating R-factors(gt) etc. and is not relevant to the choice of reflections for refinement. R-factors based on F^2^ are statistically about twice as large as those based on F, and R- factors based on ALL data will be even larger.

### Fractional atomic coordinates and isotropic or equivalent isotropic displacement parameters (Å^2^)

**Table:**

	*x*	*y*	*z*	*U*_iso_*/*U*_eq_	
N1A	0.66559 (14)	0.25954 (9)	0.45740 (10)	0.0571 (4)	
N2A	0.73238 (19)	0.22213 (10)	0.53133 (12)	0.0631 (4)	
O1A	0.63038 (14)	0.15700 (7)	0.36927 (10)	0.0709 (4)	
O2A	0.56996 (11)	0.34643 (6)	0.31969 (8)	0.0561 (3)	
C1A	0.56634 (15)	0.27126 (9)	0.30302 (11)	0.0524 (4)	
C2A	0.51543 (15)	0.25509 (10)	0.21547 (12)	0.0562 (4)	
C3A	0.48558 (15)	0.32498 (10)	0.17160 (12)	0.0556 (4)	
C4A	0.43569 (18)	0.34786 (13)	0.08226 (14)	0.0717 (6)	
H4AA	0.4123	0.3136	0.0356	0.086*	
C5A	0.42246 (19)	0.42181 (14)	0.06571 (16)	0.0810 (6)	
H5AA	0.3910	0.4377	0.0065	0.097*	
C6A	0.4547 (2)	0.47400 (13)	0.13506 (18)	0.0801 (6)	
H6AA	0.4419	0.5238	0.1218	0.096*	
C7A	0.50514 (18)	0.45342 (12)	0.22282 (15)	0.0685 (5)	
H7AA	0.5279	0.4879	0.2694	0.082*	
C8A	0.51987 (15)	0.37872 (10)	0.23764 (12)	0.0550 (4)	
C9A	0.62203 (16)	0.22390 (10)	0.37883 (12)	0.0534 (4)	
C10A	0.4931 (2)	0.18129 (11)	0.17137 (16)	0.0808 (6)	
H10D	0.4948	0.1443	0.2198	0.121*	
H10E	0.5557	0.1710	0.1291	0.121*	
H10F	0.4144	0.1810	0.1367	0.121*	
N1B	0.83956 (14)	0.29089 (8)	0.71808 (11)	0.0569 (4)	
N2B	0.82765 (19)	0.36857 (9)	0.71889 (12)	0.0633 (4)	
O1B	0.92755 (12)	0.28638 (7)	0.86711 (9)	0.0695 (4)	
O2B	0.83348 (11)	0.14361 (6)	0.70444 (8)	0.0566 (3)	
C1B	0.87755 (15)	0.17365 (9)	0.79039 (11)	0.0507 (4)	
C2B	0.90239 (15)	0.12155 (10)	0.85708 (13)	0.0560 (4)	
C3B	0.87230 (16)	0.05251 (10)	0.81085 (14)	0.0609 (5)	
C4B	0.8743 (2)	−0.02149 (13)	0.8389 (2)	0.0897 (7)	
H4BA	0.8995	−0.0349	0.9005	0.108*	
C5B	0.8378 (3)	−0.07389 (13)	0.7722 (3)	0.1080 (10)	
H5BA	0.8385	−0.1234	0.7897	0.130*	
C6B	0.8001 (3)	−0.05510 (13)	0.6802 (2)	0.0974 (8)	
H6BA	0.7776	−0.0923	0.6371	0.117*	
C7B	0.7950 (2)	0.01714 (11)	0.65068 (18)	0.0770 (6)	
H7BA	0.7685	0.0302	0.5892	0.092*	
C8B	0.83170 (16)	0.06907 (10)	0.71809 (14)	0.0587 (4)	
C9B	0.88443 (15)	0.25460 (10)	0.79526 (12)	0.0527 (4)	
C10B	0.94992 (19)	0.13066 (13)	0.95756 (14)	0.0740 (6)	
H10A	0.9272	0.1784	0.9798	0.111*	
H10B	1.0376	0.1263	0.9622	0.111*	
H10C	0.9155	0.0932	0.9955	0.111*	
N1C	0.69717 (14)	0.46541 (9)	0.57073 (10)	0.0594 (4)	
N2C	0.64389 (18)	0.42386 (9)	0.49354 (12)	0.0623 (4)	
O1C	0.64284 (13)	0.57510 (7)	0.50655 (9)	0.0699 (4)	
O2C	0.79491 (11)	0.52731 (6)	0.72827 (8)	0.0567 (3)	
C1C	0.75346 (15)	0.57452 (9)	0.65594 (11)	0.0506 (4)	
C2C	0.77132 (16)	0.64592 (9)	0.67945 (13)	0.0531 (4)	
C3C	0.82728 (16)	0.64574 (10)	0.77463 (13)	0.0554 (4)	
C4C	0.86680 (19)	0.69945 (12)	0.84080 (16)	0.0729 (6)	
H4CA	0.8602	0.7494	0.8261	0.088*	
C5C	0.9156 (2)	0.67689 (15)	0.92799 (16)	0.0819 (7)	
H5CA	0.9427	0.7122	0.9723	0.098*	
C6C	0.92547 (19)	0.60330 (15)	0.95160 (15)	0.0834 (7)	
H6CA	0.9585	0.5901	1.0115	0.100*	
C7C	0.88740 (19)	0.54871 (13)	0.88830 (14)	0.0730 (5)	
H7CA	0.8938	0.4989	0.9037	0.088*	
C8C	0.83918 (15)	0.57222 (10)	0.80065 (12)	0.0562 (4)	
C9C	0.69353 (15)	0.53881 (9)	0.57113 (11)	0.0512 (4)	
C10C	0.7425 (2)	0.71305 (11)	0.62159 (15)	0.0730 (6)	
H10G	0.7081	0.6987	0.5601	0.110*	
H10H	0.8162	0.7408	0.6153	0.110*	
H10I	0.6846	0.7429	0.6521	0.110*	
H1N1	0.6655 (17)	0.3094 (11)	0.4641 (14)	0.063 (6)*	
H2N1	0.8052 (18)	0.2666 (11)	0.6644 (15)	0.069 (6)*	
H3N1	0.7349 (17)	0.4363 (12)	0.6145 (15)	0.069 (6)*	
H1N2	0.687 (2)	0.1863 (14)	0.5467 (18)	0.085 (8)*	
H2N2	0.792 (2)	0.2002 (13)	0.5072 (17)	0.080 (8)*	
H3N2	0.901 (2)	0.3877 (13)	0.7343 (17)	0.086 (7)*	
H4N2	0.777 (2)	0.3793 (12)	0.7671 (17)	0.078 (7)*	
H5N2	0.667 (2)	0.4472 (13)	0.4439 (18)	0.084 (7)*	
H6N2	0.560 (2)	0.4227 (12)	0.4966 (14)	0.074 (6)*	

### Atomic displacement parameters (Å^2^)

**Table:**

	*U*^11^	*U*^22^	*U*^33^	*U*^12^	*U*^13^	*U*^23^
N1A	0.0717 (9)	0.0530 (8)	0.0446 (7)	0.0021 (7)	−0.0089 (7)	−0.0002 (6)
N2A	0.0743 (11)	0.0612 (9)	0.0518 (8)	0.0026 (9)	−0.0096 (8)	0.0054 (7)
O1A	0.0983 (10)	0.0501 (7)	0.0630 (7)	−0.0067 (6)	−0.0030 (7)	−0.0019 (6)
O2A	0.0668 (7)	0.0534 (7)	0.0465 (6)	−0.0018 (5)	−0.0076 (5)	−0.0024 (5)
C1A	0.0559 (9)	0.0535 (9)	0.0471 (8)	−0.0030 (7)	−0.0009 (7)	−0.0047 (7)
C2A	0.0533 (9)	0.0648 (11)	0.0500 (9)	−0.0054 (8)	−0.0002 (7)	−0.0090 (8)
C3A	0.0489 (9)	0.0718 (11)	0.0456 (8)	0.0015 (8)	−0.0012 (7)	−0.0016 (8)
C4A	0.0622 (11)	0.1009 (16)	0.0505 (10)	0.0052 (10)	−0.0048 (8)	−0.0012 (10)
C5A	0.0694 (12)	0.1060 (18)	0.0668 (12)	0.0175 (12)	−0.0008 (10)	0.0258 (13)
C6A	0.0745 (13)	0.0802 (14)	0.0854 (15)	0.0155 (11)	0.0048 (11)	0.0208 (12)
C7A	0.0692 (12)	0.0644 (12)	0.0717 (12)	0.0061 (9)	0.0025 (9)	0.0068 (10)
C8A	0.0489 (9)	0.0647 (10)	0.0510 (9)	0.0025 (8)	0.0004 (7)	0.0040 (8)
C9A	0.0574 (10)	0.0529 (9)	0.0498 (9)	−0.0076 (7)	0.0026 (7)	−0.0030 (7)
C10A	0.0981 (16)	0.0753 (13)	0.0661 (12)	−0.0077 (12)	−0.0138 (11)	−0.0197 (10)
N1B	0.0728 (9)	0.0451 (8)	0.0501 (8)	0.0014 (6)	−0.0125 (7)	0.0022 (6)
N2B	0.0806 (12)	0.0473 (8)	0.0595 (9)	−0.0021 (8)	−0.0122 (9)	0.0034 (7)
O1B	0.0821 (9)	0.0679 (8)	0.0548 (7)	−0.0060 (6)	−0.0213 (6)	−0.0025 (6)
O2B	0.0682 (8)	0.0521 (7)	0.0482 (6)	0.0001 (5)	−0.0049 (5)	0.0029 (5)
C1B	0.0499 (8)	0.0546 (9)	0.0464 (8)	0.0024 (7)	−0.0041 (7)	0.0038 (7)
C2B	0.0494 (9)	0.0615 (10)	0.0564 (9)	0.0061 (8)	−0.0003 (7)	0.0130 (8)
C3B	0.0552 (10)	0.0550 (10)	0.0726 (11)	0.0080 (8)	0.0057 (8)	0.0115 (9)
C4B	0.0939 (17)	0.0652 (14)	0.1089 (19)	0.0150 (12)	−0.0006 (14)	0.0312 (13)
C5B	0.117 (2)	0.0477 (12)	0.159 (3)	0.0043 (13)	0.009 (2)	0.0132 (16)
C6B	0.1091 (19)	0.0569 (13)	0.126 (2)	−0.0038 (12)	0.0069 (17)	−0.0131 (14)
C7B	0.0823 (14)	0.0633 (12)	0.0856 (15)	−0.0028 (10)	0.0065 (12)	−0.0092 (11)
C8B	0.0580 (10)	0.0490 (9)	0.0695 (11)	0.0038 (7)	0.0071 (8)	0.0024 (8)
C9B	0.0515 (9)	0.0557 (9)	0.0496 (8)	0.0002 (7)	−0.0041 (7)	0.0034 (8)
C10B	0.0720 (12)	0.0882 (14)	0.0593 (11)	0.0057 (10)	−0.0118 (9)	0.0227 (10)
N1C	0.0726 (10)	0.0529 (8)	0.0498 (8)	0.0019 (7)	−0.0151 (7)	−0.0022 (7)
N2C	0.0713 (11)	0.0607 (9)	0.0524 (8)	−0.0012 (8)	−0.0128 (8)	−0.0061 (7)
O1C	0.0846 (9)	0.0649 (8)	0.0573 (7)	0.0083 (7)	−0.0151 (6)	0.0084 (6)
O2C	0.0694 (7)	0.0503 (6)	0.0485 (6)	0.0037 (5)	−0.0082 (5)	−0.0003 (5)
C1C	0.0515 (9)	0.0513 (9)	0.0483 (8)	0.0039 (7)	−0.0004 (7)	0.0017 (7)
C2C	0.0500 (9)	0.0505 (9)	0.0592 (9)	−0.0001 (7)	0.0074 (7)	−0.0004 (7)
C3C	0.0483 (9)	0.0595 (10)	0.0585 (9)	−0.0050 (7)	0.0045 (7)	−0.0090 (8)
C4C	0.0694 (12)	0.0664 (12)	0.0832 (14)	−0.0098 (10)	0.0067 (11)	−0.0193 (10)
C5C	0.0709 (13)	0.1076 (19)	0.0653 (12)	−0.0078 (12)	−0.0072 (10)	−0.0302 (12)
C6C	0.0697 (13)	0.121 (2)	0.0577 (11)	0.0053 (13)	−0.0098 (10)	−0.0120 (12)
C7C	0.0753 (12)	0.0858 (14)	0.0557 (10)	0.0078 (10)	−0.0100 (9)	−0.0016 (10)
C8C	0.0551 (9)	0.0620 (10)	0.0505 (9)	0.0020 (8)	−0.0031 (7)	−0.0083 (8)
C9C	0.0508 (9)	0.0542 (9)	0.0484 (9)	0.0046 (7)	0.0012 (7)	0.0029 (7)
C10C	0.0824 (14)	0.0541 (10)	0.0826 (14)	0.0009 (9)	0.0056 (11)	0.0101 (10)

### Geometric parameters (Å, º)

**Table:**

N1A—C9A	1.345 (2)	C3B—C4B	1.399 (3)
N1A—N2A	1.408 (2)	C4B—C5B	1.378 (4)
N1A—H1N1	0.908 (19)	C4B—H4BA	0.9300
N2A—H1N2	0.86 (3)	C5B—C6B	1.383 (4)
N2A—H2N2	0.86 (2)	C5B—H5BA	0.9300
O1A—C9A	1.224 (2)	C6B—C7B	1.374 (3)
O2A—C8A	1.380 (2)	C6B—H6BA	0.9300
O2A—C1A	1.3829 (19)	C7B—C8B	1.380 (3)
C1A—C2A	1.355 (2)	C7B—H7BA	0.9300
C1A—C9A	1.473 (2)	C10B—H10A	0.9600
C2A—C3A	1.438 (3)	C10B—H10B	0.9600
C2A—C10A	1.489 (3)	C10B—H10C	0.9600
C3A—C8A	1.384 (2)	N1C—C9C	1.331 (2)
C3A—C4A	1.405 (2)	N1C—N2C	1.419 (2)
C4A—C5A	1.367 (3)	N1C—H3N1	0.89 (2)
C4A—H4AA	0.9300	N2C—H5N2	0.87 (2)
C5A—C6A	1.392 (3)	N2C—H6N2	0.92 (2)
C5A—H5AA	0.9300	O1C—C9C	1.227 (2)
C6A—C7A	1.376 (3)	O2C—C8C	1.371 (2)
C6A—H6AA	0.9300	O2C—C1C	1.3867 (19)
C7A—C8A	1.378 (3)	C1C—C2C	1.347 (2)
C7A—H7AA	0.9300	C1C—C9C	1.477 (2)
C10A—H10D	0.9600	C2C—C3C	1.441 (3)
C10A—H10E	0.9600	C2C—C10C	1.488 (2)
C10A—H10F	0.9600	C3C—C8C	1.386 (3)
N1B—C9B	1.339 (2)	C3C—C4C	1.399 (3)
N1B—N2B	1.414 (2)	C4C—C5C	1.373 (3)
N1B—H2N1	0.93 (2)	C4C—H4CA	0.9300
N2B—H3N2	0.88 (2)	C5C—C6C	1.378 (3)
N2B—H4N2	0.93 (2)	C5C—H5CA	0.9300
O1B—C9B	1.234 (2)	C6C—C7C	1.380 (3)
O2B—C8B	1.365 (2)	C6C—H6CA	0.9300
O2B—C1B	1.389 (2)	C7C—C8C	1.382 (2)
C1B—C2B	1.350 (2)	C7C—H7CA	0.9300
C1B—C9B	1.471 (2)	C10C—H10G	0.9600
C2B—C3B	1.440 (3)	C10C—H10H	0.9600
C2B—C10B	1.490 (3)	C10C—H10I	0.9600
C3B—C8B	1.391 (3)		
			
C9A—N1A—N2A	121.28 (16)	C4B—C5B—H5BA	119.0
C9A—N1A—H1N1	124.1 (13)	C6B—C5B—H5BA	119.0
N2A—N1A—H1N1	113.9 (13)	C7B—C6B—C5B	121.6 (2)
N1A—N2A—H1N2	105.9 (16)	C7B—C6B—H6BA	119.2
N1A—N2A—H2N2	107.2 (16)	C5B—C6B—H6BA	119.2
H1N2—N2A—H2N2	103 (2)	C6B—C7B—C8B	115.8 (2)
C8A—O2A—C1A	105.61 (13)	C6B—C7B—H7BA	122.1
C2A—C1A—O2A	111.99 (15)	C8B—C7B—H7BA	122.1
C2A—C1A—C9A	131.56 (16)	O2B—C8B—C7B	125.68 (18)
O2A—C1A—C9A	116.38 (13)	O2B—C8B—C3B	109.94 (16)
C1A—C2A—C3A	105.73 (15)	C7B—C8B—C3B	124.39 (18)
C1A—C2A—C10A	128.52 (18)	O1B—C9B—N1B	122.75 (17)
C3A—C2A—C10A	125.75 (16)	O1B—C9B—C1B	121.31 (15)
C8A—C3A—C4A	118.09 (18)	N1B—C9B—C1B	115.92 (14)
C8A—C3A—C2A	106.57 (14)	C2B—C10B—H10A	109.5
C4A—C3A—C2A	135.32 (18)	C2B—C10B—H10B	109.5
C5A—C4A—C3A	118.3 (2)	H10A—C10B—H10B	109.5
C5A—C4A—H4AA	120.9	C2B—C10B—H10C	109.5
C3A—C4A—H4AA	120.9	H10A—C10B—H10C	109.5
C4A—C5A—C6A	121.81 (19)	H10B—C10B—H10C	109.5
C4A—C5A—H5AA	119.1	C9C—N1C—N2C	121.54 (14)
C6A—C5A—H5AA	119.1	C9C—N1C—H3N1	126.9 (13)
C7A—C6A—C5A	121.3 (2)	N2C—N1C—H3N1	111.5 (13)
C7A—C6A—H6AA	119.4	N1C—N2C—H5N2	103.8 (16)
C5A—C6A—H6AA	119.4	N1C—N2C—H6N2	109.3 (13)
C6A—C7A—C8A	116.1 (2)	H5N2—N2C—H6N2	113 (2)
C6A—C7A—H7AA	122.0	C8C—O2C—C1C	105.44 (13)
C8A—C7A—H7AA	122.0	C2C—C1C—O2C	112.09 (15)
C7A—C8A—O2A	125.48 (17)	C2C—C1C—C9C	132.11 (16)
C7A—C8A—C3A	124.43 (17)	O2C—C1C—C9C	115.72 (14)
O2A—C8A—C3A	110.09 (15)	C1C—C2C—C3C	105.88 (15)
O1A—C9A—N1A	122.86 (16)	C1C—C2C—C10C	128.88 (17)
O1A—C9A—C1A	121.78 (15)	C3C—C2C—C10C	125.23 (16)
N1A—C9A—C1A	115.33 (15)	C8C—C3C—C4C	118.17 (18)
C2A—C10A—H10D	109.5	C8C—C3C—C2C	106.07 (15)
C2A—C10A—H10E	109.5	C4C—C3C—C2C	135.75 (18)
H10D—C10A—H10E	109.5	C5C—C4C—C3C	118.6 (2)
C2A—C10A—H10F	109.5	C5C—C4C—H4CA	120.7
H10D—C10A—H10F	109.5	C3C—C4C—H4CA	120.7
H10E—C10A—H10F	109.5	C4C—C5C—C6C	121.7 (2)
C9B—N1B—N2B	120.72 (15)	C4C—C5C—H5CA	119.1
C9B—N1B—H2N1	122.5 (12)	C6C—C5C—H5CA	119.1
N2B—N1B—H2N1	116.3 (12)	C5C—C6C—C7C	121.4 (2)
N1B—N2B—H3N2	108.1 (15)	C5C—C6C—H6CA	119.3
N1B—N2B—H4N2	105.9 (14)	C7C—C6C—H6CA	119.3
H3N2—N2B—H4N2	108 (2)	C6C—C7C—C8C	116.2 (2)
C8B—O2B—C1B	105.74 (13)	C6C—C7C—H7CA	121.9
C2B—C1B—O2B	112.32 (16)	C8C—C7C—H7CA	121.9
C2B—C1B—C9B	131.15 (16)	O2C—C8C—C7C	125.56 (18)
O2B—C1B—C9B	116.47 (13)	O2C—C8C—C3C	110.51 (15)
C1B—C2B—C3B	105.15 (16)	C7C—C8C—C3C	123.92 (18)
C1B—C2B—C10B	129.11 (18)	O1C—C9C—N1C	123.01 (16)
C3B—C2B—C10B	125.75 (17)	O1C—C9C—C1C	121.52 (16)
C8B—C3B—C4B	118.2 (2)	N1C—C9C—C1C	115.47 (14)
C8B—C3B—C2B	106.85 (16)	C2C—C10C—H10G	109.5
C4B—C3B—C2B	134.9 (2)	C2C—C10C—H10H	109.5
C5B—C4B—C3B	117.9 (2)	H10G—C10C—H10H	109.5
C5B—C4B—H4BA	121.0	C2C—C10C—H10I	109.5
C3B—C4B—H4BA	121.0	H10G—C10C—H10I	109.5
C4B—C5B—C6B	122.0 (2)	H10H—C10C—H10I	109.5
			
C8A—O2A—C1A—C2A	−0.71 (19)	C1B—O2B—C8B—C7B	−179.46 (19)
C8A—O2A—C1A—C9A	176.44 (14)	C1B—O2B—C8B—C3B	0.71 (19)
O2A—C1A—C2A—C3A	0.9 (2)	C6B—C7B—C8B—O2B	−179.66 (19)
C9A—C1A—C2A—C3A	−175.65 (18)	C6B—C7B—C8B—C3B	0.2 (3)
O2A—C1A—C2A—C10A	−178.90 (18)	C4B—C3B—C8B—O2B	178.62 (17)
C9A—C1A—C2A—C10A	4.5 (3)	C2B—C3B—C8B—O2B	−0.7 (2)
C1A—C2A—C3A—C8A	−0.80 (19)	C4B—C3B—C8B—C7B	−1.2 (3)
C10A—C2A—C3A—C8A	179.05 (18)	C2B—C3B—C8B—C7B	179.49 (18)
C1A—C2A—C3A—C4A	177.5 (2)	N2B—N1B—C9B—O1B	−6.8 (3)
C10A—C2A—C3A—C4A	−2.7 (4)	N2B—N1B—C9B—C1B	172.22 (16)
C8A—C3A—C4A—C5A	−0.7 (3)	C2B—C1B—C9B—O1B	6.4 (3)
C2A—C3A—C4A—C5A	−178.8 (2)	O2B—C1B—C9B—O1B	−176.64 (16)
C3A—C4A—C5A—C6A	−1.2 (3)	C2B—C1B—C9B—N1B	−172.58 (18)
C4A—C5A—C6A—C7A	2.1 (3)	O2B—C1B—C9B—N1B	4.4 (2)
C5A—C6A—C7A—C8A	−0.8 (3)	C8C—O2C—C1C—C2C	0.82 (19)
C6A—C7A—C8A—O2A	179.05 (18)	C8C—O2C—C1C—C9C	−176.23 (15)
C6A—C7A—C8A—C3A	−1.2 (3)	O2C—C1C—C2C—C3C	−0.7 (2)
C1A—O2A—C8A—C7A	179.92 (17)	C9C—C1C—C2C—C3C	175.70 (18)
C1A—O2A—C8A—C3A	0.17 (18)	O2C—C1C—C2C—C10C	178.58 (17)
C4A—C3A—C8A—C7A	2.0 (3)	C9C—C1C—C2C—C10C	−5.0 (3)
C2A—C3A—C8A—C7A	−179.37 (17)	C1C—C2C—C3C—C8C	0.3 (2)
C4A—C3A—C8A—O2A	−178.24 (16)	C10C—C2C—C3C—C8C	−179.00 (17)
C2A—C3A—C8A—O2A	0.39 (19)	C1C—C2C—C3C—C4C	−178.5 (2)
N2A—N1A—C9A—O1A	4.9 (3)	C10C—C2C—C3C—C4C	2.1 (4)
N2A—N1A—C9A—C1A	−173.31 (17)	C8C—C3C—C4C—C5C	0.2 (3)
C2A—C1A—C9A—O1A	0.9 (3)	C2C—C3C—C4C—C5C	178.9 (2)
O2A—C1A—C9A—O1A	−175.59 (16)	C3C—C4C—C5C—C6C	−0.5 (3)
C2A—C1A—C9A—N1A	179.11 (18)	C4C—C5C—C6C—C7C	0.4 (3)
O2A—C1A—C9A—N1A	2.6 (2)	C5C—C6C—C7C—C8C	0.0 (3)
C8B—O2B—C1B—C2B	−0.49 (19)	C1C—O2C—C8C—C7C	178.43 (18)
C8B—O2B—C1B—C9B	−178.00 (14)	C1C—O2C—C8C—C3C	−0.59 (19)
O2B—C1B—C2B—C3B	0.1 (2)	C6C—C7C—C8C—O2C	−179.19 (18)
C9B—C1B—C2B—C3B	177.12 (17)	C6C—C7C—C8C—C3C	−0.3 (3)
O2B—C1B—C2B—C10B	−179.60 (18)	C4C—C3C—C8C—O2C	179.28 (16)
C9B—C1B—C2B—C10B	−2.6 (3)	C2C—C3C—C8C—O2C	0.2 (2)
C1B—C2B—C3B—C8B	0.4 (2)	C4C—C3C—C8C—C7C	0.2 (3)
C10B—C2B—C3B—C8B	−179.95 (18)	C2C—C3C—C8C—C7C	−178.86 (18)
C1B—C2B—C3B—C4B	−178.8 (2)	N2C—N1C—C9C—O1C	1.3 (3)
C10B—C2B—C3B—C4B	0.9 (3)	N2C—N1C—C9C—C1C	−179.64 (17)
C8B—C3B—C4B—C5B	1.1 (3)	C2C—C1C—C9C—O1C	−3.4 (3)
C2B—C3B—C4B—C5B	−179.9 (2)	O2C—C1C—C9C—O1C	172.87 (16)
C3B—C4B—C5B—C6B	0.1 (4)	C2C—C1C—C9C—N1C	177.44 (18)
C4B—C5B—C6B—C7B	−1.2 (4)	O2C—C1C—C9C—N1C	−6.2 (2)
C5B—C6B—C7B—C8B	1.0 (4)		

### Hydrogen-bond geometry (Å, º)

**Table:**

*D*—H···*A*	*D*—H	H···*A*	*D*···*A*	*D*—H···*A*
N1*A*—H1*N*1···N2*C*	0.91 (2)	2.13 (2)	3.034 (2)	171.4 (17)
N1*B*—H2*N*1···N2*A*	0.93 (2)	2.15 (2)	3.081 (2)	173.1 (17)
N1*C*—H3*N*1···N2*B*	0.89 (2)	2.12 (2)	3.016 (2)	179 (3)
N2*A*—H2*N*2···O1*B*^i^	0.86 (2)	2.58 (2)	3.275 (2)	140 (2)
N2*B*—H4*N*2···O1*A*^ii^	0.93 (2)	2.33 (2)	3.176 (2)	150.4 (18)
N2*C*—H6*N*2···O1*C*^iii^	0.92 (2)	2.22 (2)	3.137 (2)	175.5 (17)
C6*A*—H6*AA*···O1*A*^iv^	0.93	2.55	3.444 (3)	162
C5*C*—H5*CA*···O1*B*^v^	0.93	2.59	3.333 (3)	137

Symmetry codes: (i) *x*, −*y*+1/2, *z*−1/2; (ii) *x*, −*y*+1/2, *z*+1/2; (iii) −*x*+1, −*y*+1, −*z*+1; (iv) −*x*+1, *y*+1/2, −*z*+1/2; (v) −*x*+2, −*y*+1, −*z*+2.
